# What do young doctors know of palliative care; how do they expect the concept to work? A ‘palliative care’ knowledge and opinion survey among young doctors

**DOI:** 10.1186/s13104-019-4462-2

**Published:** 2019-07-16

**Authors:** G. V. M. C. Fernando, S. Prathapan

**Affiliations:** 1 National Centre for Primary Care and Allergy Research, University of Sri Jayewardenepura, Gangodawila, Nugegoda, Sri Lanka; 2 Department of Family Medicine, Faculty of Medical Sciences, University of Sri Jayewardenepura, Gangodawila, Nugegoda, Sri Lanka; 3 Department of Community Medicine, Faculty of Medical Sciences, University of Sri Jayewardenepura, Gangodawila, Nugegoda, Sri Lanka

**Keywords:** Medical student and resident education, Palliative care, Palliative medicine, Education, Medical, Undergraduate

## Abstract

**Objectives:**

Discipline of palliative care is still evolving in developed parts of the world while it remains at an infantile stage in Sri Lanka which has not been formally assessed as of today. We aimed at evaluating the level of palliative care knowledge and opinions among young medical graduates. A descriptive cross-sectional study was carried out among pre-residency medical graduates of Sri Lanka through a social media based online survey. The pre-tested questionnaire assessed the level of knowledge on general principles, service organization, clinical management and ethical considerations while it also evaluated their opinions.

**Results:**

Response rate was 35.8% (n = 351). The average score among the respondents was 37.25% [standard deviation (SD) = 11.975]. Specific knowledge on “general principles” was adequate (score ≥ 50%) with an average of 62.61%, SD = 24.5 while “ethics” was observed to be the area with the poorest knowledge (average score = 19.55%, SD = 22). Average scores for “service organization” and “managerial aspects” were 34.54%, SD = 17.6 and 32.26%, SD = 22.3, respectively. The majority (> 90%) believed that de-novo establishment of hospice, hospital and community-based palliative services would sustainably improve holistic patient care. Measures must be taken to optimize basic palliative care knowledge among the undergraduates in view of achieving Universal Health Coverage in the long term.

**Electronic supplementary material:**

The online version of this article (10.1186/s13104-019-4462-2) contains supplementary material, which is available to authorized users.

## Introduction

Palliative care is an approach that aims to improve the quality of life of patients, and their families facing the physical, psychosocial and spiritual problems associated with life-threatening illnesses [1].

Department of Census and Statistics of Sri Lanka declared in 2016 through a press release that over the decade preceding year 2011, life expectancy has risen considerably [2]. With the advancement of diagnostic technologies, most terminal diseases are revealed at their prodromal stages, resulting in a rise in the population living with life-limiting illnesses. A study conducted in the United Kingdom estimated a minimum of 63.03% of all deaths need palliative care [3].

A systematic review of international literature on teaching and learning in palliative care within medical undergraduate curricula pointed out the lack of consistency and fragmentation of teaching in the discipline, owing to lack of efficient inter-departmental collaborations. The importance of devising an integrated curriculum for palliative care with a strong emphasis on a multidisciplinary approach was recommended [4].

The discipline of palliative care thrives at infancy in Sri Lanka. Relevant specialists and published evidence on the discipline lack in the country as of today. Nevertheless, palliative services are on a gradual incline [5]. In such a background it is worthwhile exploring the knowledgeability of young doctors on ‘Palliative Medicine’ to cater the ever-growing palliative care needs [6]. A PubMed search on undergraduate palliative care/medicine education only revealed one study that was carried out among medical students of the Faculty of Medicine, University of Colombo. The investigator found out that only 22% of the students were familiar with the concept of palliative care. While 76% of them held that they had inadequate knowledge to manage symptoms in a dying patient, the majority also wished to have palliative care included in the undergraduate medical curriculum [7].

The current survey aimed at evaluation of the knowledge and opinions on palliative care among medical graduates who awaited to commence residency in 2017. It was expected to provide valuable information for the academics, healthcare staff and policymakers to align their vision accordingly.

## Main text

### Methods

Considering the 1186 candidates who have reportedly faced the Bachelor of Medicine, Bachelor of Surgery (MBBS) examination in the year 2017 in Sri Lanka, the minimum sample size calculated to have sufficient representation and statistical power was 291 (for a Confidence Interval of 95% with the Standard Error of Mean set at 5%) [8, 9].

A descriptive cross-sectional study was designed (#07/17, Ethics Review Committee, Faculty of Medical Sciences, University of Sri Jayewardenepura, Sri Lanka) to evaluate medical professionals graduated during the year 2017 from 8 medical faculties of Sri Lanka. (Kotelawala Defence University had not produced its debut batch by this time). The undergraduates who have not officially completed the degree (owing to any reason) were excluded.

### Assessment

There was no consideration to examine the palliative content in the undergraduate curricula initially since there are no registered academic departments (under the University Grants Commission of Sri Lanka), relevant specialists or defined academic programmes concerning palliative care in any of the medical schools in the country. The study intended to ascertain the level of knowledge of the current graduates, in the hope that its results of will be instrumental in devising an academic programme in the future to address the knowledge gap as appropriate.

An online self-administered questionnaire was generated via Google Forms [10]. In the absence of a validated questionnaire specifically devised to assess the level of knowledge on palliative care as of the time, the essential core knowledge was assessed as per the endorsements for undergraduate medical education by the European Association for Palliative Care (2007) [11]. The validity of the questionnaire was ensured by content validity and face validity. The questionnaire was developed after an intensive literature search and then it was reviewed by a specialist (foreign) in the field of Palliative care and a local specialist in medical education. Amendments in the questions were made by common consensus among the aforesaid specialists. Reliability was ensured during the pre-test and was retested again in two weeks.

The questionnaire was pre-tested on 10 doctors randomly selected from the Facebook social media account of the principle-investigator (PI), employed at institutions under the Ministry of Health, regardless of their current work station or age. As per the results of the pilot study, one question was substituted (in the ‘clinical management’ domain) considering the 80% incorrect response rate, while some other questions were rephrased prior to the proper dissemination of the questionnaires via social media between 7th March to 15th August 2017.

The PI contacted the student representatives of each medical faculty over the telephone. Facebook and/or WhatsApp were recognized to be the most popular social media platforms where the young doctors utilized for networking with the fellow colleagues of the same batch. The individual social media accounts of the graduates on these groups were retrieved from these representatives, through which the form was shared individually and directly by the PI. Non-responders were reminded twice more at monthly intervals from the initial contact. The messages included a request to circulate the form among the fellow batch-mates thus employing ‘snow balling sampling method [12] thereon.

The questionnaire (Additional file 1: Appendix 1) was structured to collect the demographic details of the participant, knowledge under 4 separate domains; (basic principles, service organization, clinical management and ethical considerations) each domain containing 4 questions, followed by their opinions. Their opinions were assessed through ten pre-formulated statements. In response to them, the participants were expected to mark their level of agreement on a scale (‘disagree’/‘neutral’/‘agree’).

Logging into personal accounts, reading the information followed by checking the “tick-box” was considered equivalent to informed consent. Duplicate submissions from the same account were blocked.

### Statistical analysis

Data were analysed using SPSS 20 software. The average percentage scores relevant to the level of knowledge of the doctors were assessed overall and under each domain. A score of 50% or more was regarded to be indicative of adequate knowledge for medical graduates by the aforementioned specialists.

The existence of a significant correlation of the knowledge levels with sociodemographic factors was assessed using Pearson *χ*^2^ (Chi square) adopting a significance level of 5% and a confidence level of 95%.

The level-of-agreement categories (agree, neutral or disagree) of the participants regarding each statement were assessed in percentages and contrasted with current (albeit conflicting) evidence.

### Results

Of the total intake of 1215 graduates to all state medical faculties in the relevant academic year [13], 1186 had faced the final MBBS examination out of whom 981 have subscribed to social media groups of the relevant batch. Response rate was 35.8% (n = 351) (Additional file 2: Appendix 2: Data Sheet containing all retrieved data). The demographic details of the respondents are illustrated in Table 1.

**Table 1:** Sociodemographic characteristics of the population assessed

Gender	Proportion (number and percentage)
Male	146 (42%)
Female	205 (58%)
Age	Value/s
Mean age (years)	25.81
Age range (years)	23-28
Standard deviation (years)	0.87
Religion	Proportion (number and percentage)
Buddhist	275 (78.35%)
Hindu	29 (8.26%)
Islam	23 (6.55%)
Catholic	14 (3.99%)
Christian	6 (1.71%)
Atheist	4 (1.14%)
Ethnicity	Proportion (number and percentage)
Sinhalese	293 (83.48%)
Tamil	33 (9.40%)
Moor	23 (6.55%)
Bhutanese	2 (0.56%)
Employment	Proportion (number and percentage)
Temporary demonstrator	150 (42.74%)
Research assistant	83 (23.65%)
Locum medical officer	54 (15.38%)
Not employed	58 (16.52%)
Other	6 (1.70%)
Medical school	Proportion (number and percentage)
Colombo	63 (17.9%)
Eastern	18 (5.1%)
Jaffna	18 (5.1%)
Peradeniya	68 (19.3%)
Ragama	44 (12.5%)
Rajarata	26 (7.4%)
Ruhuna	25 (7.1%)
Sri Jayewardenepura	89 (25.3)

#### Basic knowledge on ‘Palliative Care’

The average score for the entire knowledge section was 37.23% (SD = 11.975%). The score distribution is illustrated in Table 2.

**Table 2:** Illustration of knowledge scores

Domains	Percentage of “correct” responses and standard deviation (SD)	Percentage of “incorrect” responses (%)	Percentage of “uncertain” responses (%)	Percentage of respondents who scored > 50% (%)
A. General principles	62.60% (SD = 24.5%)	25.57	11.82	82.9
B. Service organization	34.54% (SD = 17.6)	29.27	36.18	40.7
C. Clinical management	32.26% (SD = 22.3)	38.96	28.77	36.8
D. Ethical considerations	19.53% (SD = 20)	56.54	23.93	20.8
Overall	37.23% (SD = 11.975)	37.59	25.18	17.7

Gender, age, the medical school of graduation, employment status, religion and ethnicity did not have a significant effect on their scores. The analysis revealed a significant discrepancy only between the knowledge levels between the service organization and clinical management domains (p = 0.019) in individual respondents.

#### Opinions regarding palliative care

The results are illustrated in Table 3 in terms of the level of consonance with each statement.

**Table 3:** Detailed analysis of individual opinions of the doctors

Statement	Agree	Neutral	Disagree
*“The number of patients in need for palliative care is on the rise”*	298 (85%)	37 (10%)	16 (5%)
*“All dying patients should receive palliative care”*	169 (48%)	82 (23%)	100 (29%)
*“Patients should be informed about their prognosis be it favourable or otherwise”*	238 (68%)	89 (25%)	24 (7%)
*“Family/Loved ones should decide which details about the illness the patient should receive”*	108 (31%)	102 (29%)	141 (40%)
*“Steroids improve the quality of life of palliative patients”*	140 (40%)	164 (47%)	47 (13%)
*“There would be no difference in the way a patient with a terminal diagnosis is approached by a Palliative Specialist as opposed to other specialists”*	49 (14%)	65 (18%)	237 (68%)
*“More hospices should be established in Sri Lanka”*	312 (89%)	34 (10%)	5 (1%)
*“Introduction of a “Hospital Palliative Care Team/Unit” will improve patient care in Sri Lankan hospitals”*	334 (95%)	16 (5%)	1 (0%)
*“Home based palliative care is much required by Sri Lankan society”*	317 (90%)	29 (8%)	5 (2%)
*“The burden of other non*-*communicable diseases is less frequent than that of cancer”*	56 (16%)	29 (8%)	266 (76%)

#### Critical discussion

Since the study was representative of all the medical schools in Sri Lanka (with each school’s response rate 18% or above), their knowledge and opinions reflect those of all young doctors.

##### Knowledge gap

The results demonstrate a deficiency in palliative care knowledge among the medical undergraduates of Sri Lankan Universities which may reflect suboptimal education. Of the four domains assessed, the graduates lacked knowledge in clinical, organizational and ethical imperatives; nevertheless, they were sufficiently aware of ‘general principles’ (scoring 62.6%) that delineate the scope of the discipline. Hence their opinions on how to implement palliative care in the Sri Lankan context were valued. Nevertheless, the poor knowledge on ethics that is integrated within the core of palliative approach to care, must be explored for its causation and addressed accordingly [14, 15].

This calls for an urgent need to integrate palliative care efficiently into the undergraduate academic curricula to meet the ever-rising demand for it in the country with inter-disciplinary liaison between all clinical departments. Emphasis should be concentrated on areas with weaker knowledge; especially ethics which form the core of palliative care 11/07/2019 11:02:00.

##### Opinions held

The majority (85%) of the young doctors felt a potential incline in the number of patients with palliative needs, which keeps on a par with the rising elderly population surviving terminal illnesses [2, 16–18]. Traditionally palliative care caters for life-limiting chronic illnesses. But there is emerging evidence that application of the same principles of management into acute care (e.g. trauma) settings may be associated with improved patient outcomes [19, 20]. Forty-eight per cent believed that *“all dying patients should receive palliative care”* regardless of acute or insidious progression into the terminal illness [21].

*“Patients should be informed about their prognosis be it favourable or otherwise”*

*“Loved ones should decide which details about the illness the patient should receive”*

Above two statements from the questionnaire relate to areas of controversy. Where the truth-telling practice is predominant in the western world, ‘hiding bitter truth’ was particularly found to be justifiable in many eastern cultures [22, 23]. Hence it is suggested by recent literature to honour the patient’s autonomy in receiving information catered to his wishes [24]. However, colluding with the patient in terms of illness-related facts such as prognosis is regarded to be detrimental overall with current evidence. It is found to lead to emotional distress, demoralization and also render them unable to formulate care plans for the future or attend to unfinished business before it is too late [25]. In our study, 68% believed it was appropriate to reveal the prognostic information to the patients in spite of the content, while 31% believed that the next-of-kin should censor the information reaching the patient.

Forty per cent of the doctors believed that steroids enhance the overall quality of life of patients, 47% remained neutral while the rest disagreed to this statement. Corticosteroids are being prescribed for many non-specific symptoms such as anorexia-cachexia symptoms, nausea, pain and breathlessness [26]. Steroids are expected to improve appetite, resolve fatigue and oedematous collections in tissues, thus assisting to improve quality of life [27, 28]. Role of steroids in addressing specific causes such as neurological complications (e.g. spinal cord compression), soft tissue infiltrations (e.g. abdominal tumours) etc. have only yielded weak evidence so far [26]. Simultaneously, steroids also contribute significantly to the adverse effect burden [26, 29, 30]. Therefore, the net benefits over harms of steroids on palliative patients is an area pending further investigations.

The uniqueness of the discipline of palliative care lies in the holistic approach to care with more emphasis on respecting clinical ethics [31, 32]. Theory on how palliative care should be practised does not necessarily dictate or oblige healthcare providers to comply with aspects that preserve ethics. This is partially attributable to the potential psychological ailments resulted such as compassion fatigue, moral distress and burnout [33, 34] among the health staff. However, the expected difference between the care approach of palliative physicians as opposed to others was seemingly apparent to 68% of the young doctors.

Globally, the facilities available for non-cancer patients to receive palliative care remain suboptimal at present [35]. Albeit the spectrum of conflicting evidence from international studies [36–39], 76% of the respondents have perceived that the symptom and healthcare burden of non-malignant diseases outweigh those of cancer.

Despite the general consensus that palliative care improves quality of life of patients at home, hospice and hospital levels, there are controversies with regards to which levels are most appropriate for the particular countries or settings depending on the financial resources, existing health infrastructure and human resources with necessary expertise etc. [40–44] The majority (> 90%) believed that de-novo establishment of each or either of hospice, hospital-based or home-based palliative care services would sustainably improve overall patient care. These are important areas to be objectively explored with future studies that will provide a base for appropriate health recourse allocation at the very inception of the establishment of palliative care facilities in the country.

## Limitations

The absence of a standardized, validated questionnaire to formally evaluate the level of palliative care knowledge among (undergraduate) medical doctors as of this time was the most significantly perceived limitation of the study. Graduates who have not subscribed to or not active on social media groups were technically inaccessible to be evaluated. Owing to the convenience sampling techniques employed (voluntary participation and ‘snow balling’), more doctors graduated from the PI’s own University (of employment) were noted to respond more frequently as opposed to the remainder. Therefore, all medical faculties were not equally represented and all doctors did not have an equal chance of being selected.

There would have been participants who availed themselves on-line study material while participation. It was also noted that, out that out of the doctors among whom the survey was distributed, those who had a particular interest for the discipline of palliative care and/or those who were enthusiastic academics spent time to take part as opposed to the remainder.

## Additional files

**Additional file 1: Appendix 1.** Questionnaire. The questionnaire used to collect data through online survey platform.

**Additional file 2: Appendix 2.** Data Sheet. All data retrieved from the online survey platform.

## Data Availability

The datasets generated during the current study have been also available from the corresponding author upon reasonable request.

## Abbreviations

MBBS: Bachelor of Medicine, Bachelor of Surgery
PI: principle investigator
SD: standard deviation
*χ*^2^: Chi square
