# Supplementary material for: What do young doctors know of palliative care; how do they expect the concept to work? A ‘palliative care’ knowledge and opinion survey among young doctors
Source: BMC Res Notes. 2019 Jul 16;12:419. doi: 10.1186/s13104-019-4462-2 (PMC6636058; doi:10.1186/s13104-019-4462-2)
Supplement: Supplementary file 1 — Additional file 1: Appendix 1. Questionnaire. The questionnaire used to collect data through online survey platform. [file 13104_2019_4462_MOESM1_ESM.docx]

**Sri Lankan pre-internship Medical Doctors’ Knowledge and Opinions on Palliative Care and End of Life issues. (available at: https://docs.google.com/forms/u/1/d/e/1FAIpQLSc_wHB7msx-tsPFDLsRlIWHEly4KU3se9pfvhPKXR7FgEOD_A/viewform)**

**Section A: Demographic details** of the participant (Will be kept confidential)

| Age at at 31^st^ December 2016 |  |
| --- | --- |

| Sex | M | F | Prefer not to say |
| --- | --- | --- | --- |

| Employment (if any) | Temporary Demonstrator/ Research Assistant/ Locum Medical Officer/ Temporary Lecturer/ Other/ Not employed |
| --- | --- |

| Religion (Practiced) | Buddhist/ Hindu/ Islam/ Catholic/ Christian/Atheist/ Other |
| --- | --- |

| Ethnicity (At birth) | Sinhalese/ Tamil/ Moor/ Other |
| --- | --- |
| Faculty Graduated | Colombo, Peradeniya, Sri Jayewardenepura, Ruhuna, Jaffna, Ragama, Eastern, Rajarata, KDU |

**Section B: Knowledge on** Palliative Care and Related Topics (True -T/False - F/Do not know - DK)

1. **General principles – Palliative Care**

|  | Is aimed at increasing the life expectancy of terminally ill patients. |
| --- | --- |
|  | May improve survival. |
|  | Bereavement support to the loved ones following the death of the patient concerned comes under the purview of palliative care. |
|  | Patients with Neurological disease, Chronic Obstructive Pulmonary Disease (COPD), Rheumatoid Arthritis may need palliative care. |

1. **Service Organization**

|  | Oncologist is the ideal coordinator of the multidisciplinary team involved. |
| --- | --- |
|  | Palliative care is provided exclusively in hospices and not tertiary healthcare institutions. |
|  | The main duty of the “Lasting Power-of-attorney” is to construct the “Advanced Care Directive”. |
|  | Pastoral care givers are involved with social domain of palliation. |

1. **Drugs and management aspects**

|  | The preferred routes of administration of drugs in the most terminal stages of life is “oral” and “rectal” (non-invasive). |
| --- | --- |
|  | Anti-convulsants may be added to the step one of WHO analgesic ladder. |
|  | Hyperkalaemia is the commonest life-threatening metabolic emergency in palliative patients. |
|  | Due to the development of tolerance the dosage for morphine for otherwise healthy adults is allowed up to a maximum of 600mg per day. |

1. **Ethical concerns**

|  | Palliative care should be incorporated into the care plan of a patient with a terminal diagnosis only after the treatments with curative intent have failed. |
| --- | --- |
|  | Although “Palliative Sedation” usually hastens death, it is not considered a serious issue in the terminal stages of a patients’ life. |
|  | “Death rattle” (noisy respiratory secretions) noticed closer to the patient’s death is one of the most distressing symptoms suffered by the patient. |
|  | Evidence shows that most patients with terminal diagnoses wishes that only their next-of-kin/family understands the prognosis. |

**Section C: Your opinions** on Palliative Care.

| **Level of agreement** | **Number** |
| --- | --- |
| Disagree | -1 |
| Neutral | 0 |
| Agree | +1 |

a. The number of patients in need for palliative care is on the rise

| -1 | 0 | +1 |
| --- | --- | --- |

b. All dying patients should receive palliative care

| -1 | 0 | +1 |
| --- | --- | --- |

c. Patients should be informed about their prognosis be it favourable or otherwise

| -1 | 0 | +1 |
| --- | --- | --- |

d. Family/Loved ones should decide which details about the illness the patient should receive.

| -1 | 0 | +1 |
| --- | --- | --- |

e. Steroids improve the quality of life of palliative patients.

| -1 | 0 | +1 |
| --- | --- | --- |

f. There would be no difference in the way a patient with a terminal diagnosis is approached by a Palliative Specialist as opposed to other specialists.

| -1 | 0 | +1 |
| --- | --- | --- |

g. More hospices should be established in Sri Lanka.

| -1 | 0 | +1 |
| --- | --- | --- |

h. Introduction of a “Hospital Palliative Care Team/Unit” will improve patient care in Sri Lankan hospitals.

| -1 | 0 | +1 |
| --- | --- | --- |

i. Home based palliative care is much required by Sri Lankan society.

| -1 | 0 | +1 |
| --- | --- | --- |

j. The burden of other non-communicable diseases is less frequent than that of cancer.

| -1 | 0 | +1 |
| --- | --- | --- |
